# How to Optimise the Collection of Patient-Reported Outcomes in the Context of a Specific Disease such as Eating Disorders

**DOI:** 10.1192/j.eurpsy.2022.70

**Published:** 2022-09-01

**Authors:** U. Schmidt

**Affiliations:** King’s College London, Institute Of Psychiatry, Psychology And Neuroscience, London, United Kingdom

**Keywords:** Anorexia nervosa, patient-reported outcomes, feedback, bulimia nervosa

## Abstract

Background: Eating disorders (EDs) are severe psychiatric disorders which, when left untreated, can lead to psychosocial impairment, physical disability and death. In the United Kingdom, many specialist ED services collect routine outcome measures (ROMs) which serve to assess illness severity, patients’ quality of life and function. The repeated collection of ROMs over the course of treatment allows for the objective evaluation of patient progress towards recovery. Recent National Health Service (NHS) guidance on adult ED care in England suggests that all services should use ROMs, not just to track progress, but also to support the achievement of collaboratively identified, person-specific recovery goals, to empower patients and inform individualised treatment. To achieve this objective, clinicians need access to psychometrically sound ROMs which can be utilised in a collaborative and person-centred manner. Traditionally, ROMs have been collected using standardised patient-reported outcome measures (PROMs), but increasingly individualised PROMs (i-PROMs) are also being developed. Methods & Findings: In this talk I will review the ‘why, what and how’ of ROMs, PROMs, I-PROMS and of associated normative and ipsative feedback on these measures in the eating disorders context. Conclusions: Use of PROMs has much to be commended both in regard to treating individual patients, at service level and also the wider health care system.

**Disclosure:**

No significant relationships.

